# *Alternaria*-derived serine protease activity drives IL-33–mediated asthma exacerbations

**DOI:** 10.1016/j.jaci.2014.02.002

**Published:** 2014-09

**Authors:** Robert J. Snelgrove, Lisa G. Gregory, Teresa Peiró, Samia Akthar, Gaynor A. Campbell, Simone A. Walker, Clare M. Lloyd

**Affiliations:** Leukocyte Biology Section, National Heart and Lung Institute, Imperial College London, London, United Kingdom

**Keywords:** *Alternaria alternata*, allergic airway disease, asthma exacerbation, protease, IL-33, AEBSF, 4-(2-Aminoethyl)benzenesulfonyl fluoride hydrochloride, ALT, *Alternaria*, BALF, BAL fluid, CAT, Cat dander, HDM, House dust mite, MCPT-1, Mast cell protease-1, MMP-9, Matrix metalloproteinase-9, PAP, Papain, PAR-2, Protease activated receptor 2, RAG, Ragweed, TRYP, Trypsin

## Abstract

**Background:**

The fungal allergen *Alternaria alternata* is implicated in severe asthma and rapid onset life-threatening exacerbations of disease. However, the mechanisms that underlie this severe pathogenicity remain unclear.

**Objective:**

We sought to investigate the mechanism whereby *Alternaria* was capable of initiating severe, rapid onset allergic inflammation.

**Methods:**

IL-33 levels were quantified in wild-type and ST2^−/−^ mice that lacked the IL-33 receptor given inhaled house dust mite, cat dander, or *Alternaria*, and the effect of inhibiting allergen-specific protease activities on IL-33 levels was assessed. An exacerbation model of allergic airway disease was established whereby mice were sensitized with house dust mite before subsequently being challenged with *Alternaria* (with or without serine protease activity), and inflammation, remodeling, and lung function assessed 24 hours later.

**Results:**

*Alternaria,* but not other common aeroallergens, possessed intrinsic serine protease activity that elicited the rapid release of IL-33 into the airways of mice through a mechanism that was dependent upon the activation of protease activated receptor-2 and adenosine triphosphate signaling. The unique capacity of *Alternaria* to drive this early IL-33 release resulted in a greater pulmonary inflammation by 24 hours after challenge relative to the common aeroallergen house dust mite. Furthermore, this *Alternaria* serine protease–IL-33 axis triggered a rapid, augmented inflammation, mucus release, and loss of lung function in our exacerbation model.

**Conclusion:**

*Alternaria-*specific serine protease activity causes rapid IL-33 release, which underlies the development of a robust T_H_2 inflammation and exacerbation of allergic airway disease.

Asthma is characterized by reversible airway obstruction as a result of a predominantly T_H_2-driven airway inflammation and pulmonary remodeling. For individuals who exhibit a genetic predisposition, asthma can be induced or exacerbated by an abnormal response to environmental stimuli, such as allergens, infectious agents, or airborne pollutants. In addition to activation of pattern recognition receptors, many allergens possess intrinsic proteolytic activities that play an important role in development of allergic airway disease, in part, by activation of protease-activated receptors (PAR). IL-33 has been associated with the development and maintenance of allergic asthma via ligation of its receptor ST2.^1^ IL-33 is located within the nucleus of Type II epithelial cells in the lung, but mechanisms that dictate its release remain ambiguous. It has been suggested that IL-33 can be released during necrosis in response to infection or trauma, and subsequently functions as an “alarmin.”^2^, ^3^ Pulmonary IL-33 expression is elevated with individuals with asthma, which correlates with asthma severity.^4^, ^5^ Modulation of the IL-33–ST2 axis in murine models of allergic airway disease has supported a prominent role for this cytokine in asthma.^6^, ^7^, ^8^, ^9^, ^10^, ^11^, ^12^ Genetic analysis also has linked polymorphisms in human IL-33–ST2 to the incidence of asthma.^13^

Severe asthma with fungal sensitization is characterized by the presence of severe asthma, fungal sensitization, and the exclusion of bronchopulmonary aspergillosis.^14^ Epidemiologic studies have identified sensitivity to fungal allergens as a prominent cause of allergic asthma.^15^ An association exists between sensitivity to the widely distributed fungus *Alternaria alternata* and asthma severity, hospital admission, and fatal asthma exacerbations.^14^, ^16^, ^17^, ^18^, ^19^, ^20^, ^21^, ^22^, ^23^ High *Alternaria* spore counts are detected in late summer and/or early autumn, where dispersion of spores is associated with thunderstorms and leads to increased morbidity and mortality.^18^, ^19^, ^24^, ^25^, ^26^ The prevalence of severe asthma with fungal sensitization has been estimated to be as frequent as 30%, although why molds are implicated in severe asthma compared with other aeroallergens has not been elucidated.

Fungal allergens, such as *Alternaria*, possess intrinsic proteolytic activities that have the potential to act as adjuvants in driving a prolonged T_H_2 inflammation.^27^, ^28^ Although the exact mechanism that defines this potential remains poorly defined, TLR4 activation has been implicated.^29^ *Alternaria*-specific serine protease activity has previously been demonstrated to elicit epithelial cell increases in intracellular calcium through protease activated receptor 2 (PAR-2) activation and to drive pulmonary inflammation.^30^ In the present study, we demonstrate that *Alternaria*-specific serine protease activity promotes the release of IL-33 in a murine model, which subsequently drives a robust release of early innate mediators and T_H_2 pulmonary inflammation. Importantly, this serine protease–mediated IL-33 release was shown to underlie *Alternaria*-driven severe exacerbations of allergic airway disease.

## Methods

### Mice

Female BALB/c mice (Charles River, Margate, United Kingdom) and ST2^−/−^ mice on a BALB/c background (a kind gift from Andrew McKenzie, MRC Laboratory of Molecular Biology, Cambridge, United Kingdom), 6 to 8 weeks old received 10 μg house dust mite (HDM) (*Dermatophagoides pteronyssinus*), *Alternaria alternata*, cat dander, or ragweed (*Ambrosia artemisiifolia*) extract (Greer, Lenoir, NC); papain or trypsin (Sigma-Aldrich, Dorset, United Kingdom); or 50 μL of vehicle, PBS intranasally. Mice were culled either 1 or 24 hours after challenge. In some experiments, the *Alternaria* extract was preincubated with either 4-(2-Aminoethyl)benzenesulfonyl fluoride hydrochloride (AEBSF) (25 mg/mL) or suramin (2 mM) (Sigma-Aldrich). FSSLYR-amide (Bachem AG, Bubendorf, Switzerland) was administered 30 minutes before allergen challenge (100 μg intraperitoneal and 12.5 μg intranasal). In the exacerbation protocol, mice were treated with either 15 μg HDM or PBS 3 times a week for 3 weeks before receiving single challenge with 10 μg *Alternaria*. All the experiments were performed in accordance with UK Home Office guidelines. Airway responsiveness was determined by direct measurements of lung function in anesthetized and tracheostomized mice 24 hours after final challenge.

### Tissue processing

Serum, BAL fluid (BALF) and lung tissue were collected.^31^ Paraffin-embedded sections (4 μm) were stained with hematoxylin and eosin and periodic acid–Schiff. Paraffin sections were stained with goat anti-mouse IL-33 (R&D Systems, Abingdon, United Kingdom) by using an avidin-biotin staining method.

### Mediator analysis

BALF was analyzed by ELISA: IL-4, IL-5 (PharMingen, Oxford, United Kingdom), IL-13, IL-33, IL-25, matrix metalloproteinase-9 (MMP-9) (R&D Systems), IL-1β (eBioscience, Hatfield, United Kingdom), and albumin (Bethyl Laboratories, Montgomery, Tex). Uric acid was measured using an Amplex red uric acid/uricase assay kit (Invitrogen, Paisley, United Kingdom). Lactate dehydrogenase was measured by using an In Vitro Toxicology Assay kit (Sigma-Aldrich). Serum mast cell protease (MCP-1) was measured by ELISA (eBioscience, Hatfield, United Kingdom). IL-33 size was determined by Western blot. MMP-9 activity was determined by using Novex 10% zymogram gelatin gels (Invitrogen). MUC5AC transcript levels were determined by quantitative PCR.

### Flow cytometric analysis

Disaggregated lung cells were restimulated with 500 ng/mL of ionomycin and 50 ng/mL of phorbol 12-myristate 13-acetate in the presence of brefeldin A (BD Pharmingen, Oxford, United Kingdom). Cells were stained for CD3, CD4, CD8, IL-13, IL-17, or IFN-γ (eBioscience). In addition, cells were stained with Ly-6G, SiglecF, CD11b, CD11c, F4/80, CD45, lineage negative cocktail (eBioscience), T1/ST2 (Morwell Diagnostics, Zurich, Switzerland), or ICOS (Biolegend, London, United Kingdom). Labeled cells were acquired on a BD Fortessa (BD Bioscience, Oxford, United Kingdom), and analyzed by using FlowJo (Treestar, Ashland, Ore).

### Statistical analysis

Data were analyzed by using Prism 4 (GraphPad Software Inc, La Jolla, Calif). Multiple comparisons were performed by using the Kruskal-Wallis test. A 2-tailed *P* value was determined by the Mann-Whitney test when comparing between 2 groups. Additional detail on the methods used in the present study are provided in this article's Online Repository at www.jacionline.org.

## Results

### *Alternaria*-specific serine protease activity drives an early IL-33–mediated inflammation

**Fig 1:**
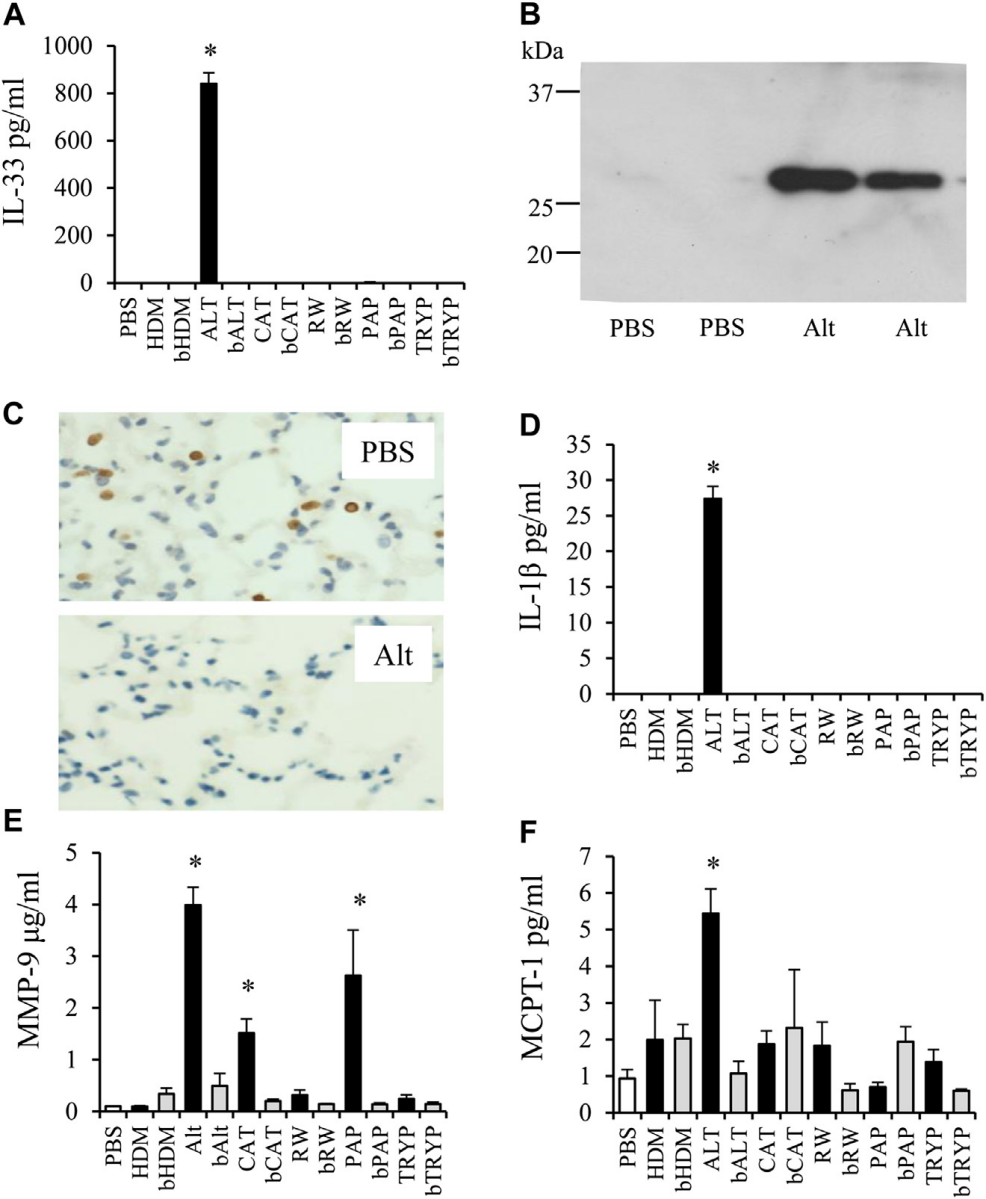
Acute allergen-induced mediator release. **A,** IL-33 levels in the BALF of mice treated with HDM, *Alternaria (ALT)*, cat dander *(CAT)*, ragweed *(RAG)*, papain *(PAP)*, or trypsin *(TRYP)*. “B” denotes boiled extract. **B,** Western blot of IL-33 protein in BALF. **C,** Immunohistochemistry, demonstrating alveolar epithelial expression of IL-33 (brown stained cells). IL-1β **(D)** and MMP-9 levels in the BALF **(E)**. **F,** Serum MCPT-1 levels. Data are presented as mean ± SEM (3 mice per treatment group). *P < .05 compared with PBS-treated mice. Representative photomicrographs are shown. Original magnification ×40. *Scale bar* = 50 μm.

**Fig E1:**
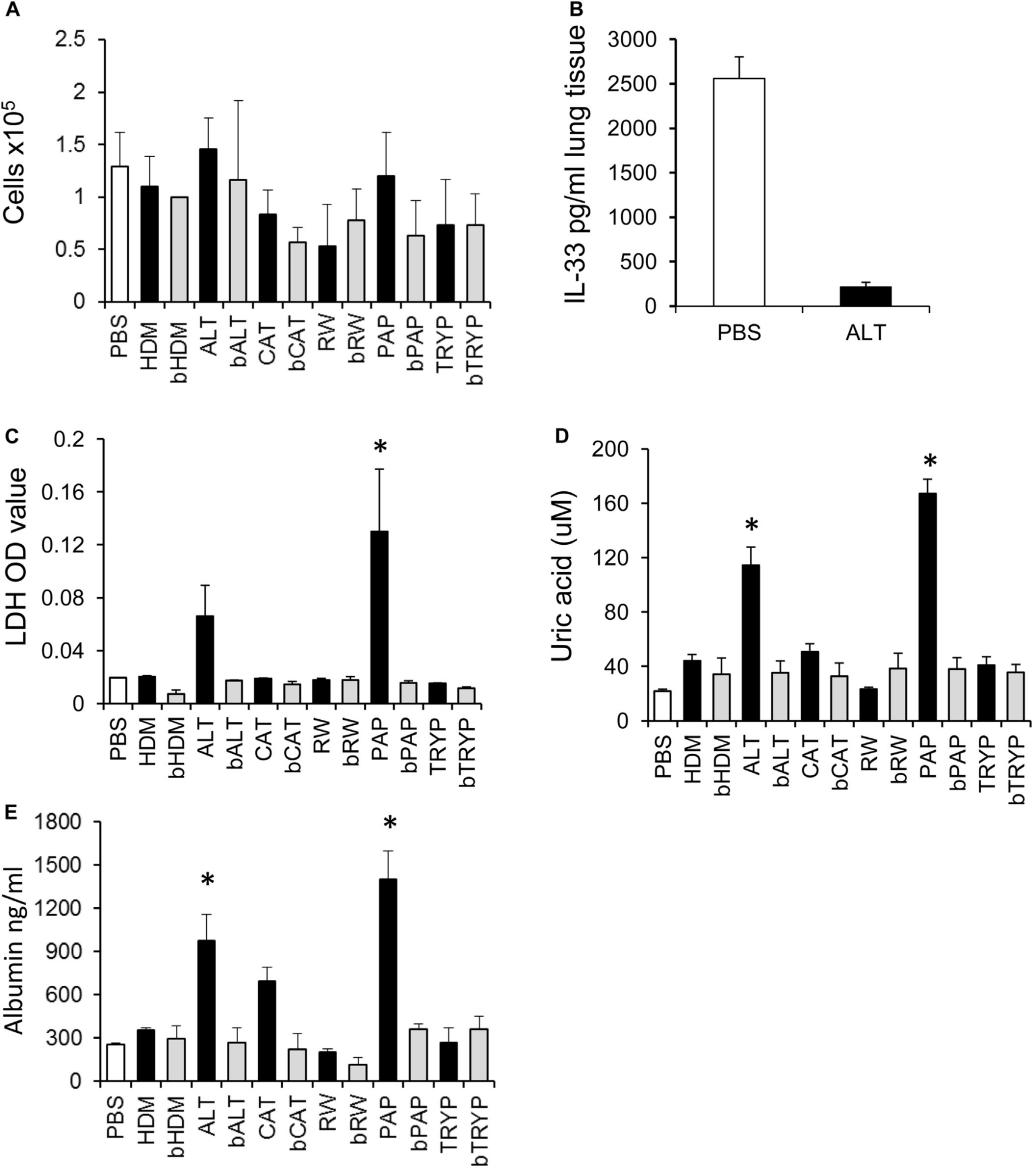
Innate mediator release in the BALF after allergen challenge. Mice were treated with extracts of either HDM, *Alternaria (ALT)*, cat dander *(CAT)*, ragweed *(RAG)*, or the proteases papain *(PAP)* or trypsin *(TRYP)*. B denotes boiled extract. **A,** Total cells. **B,** IL-33 levels in the lung 1 hour after PBS or ALT exposure. **C,** Lactate dehydrogenase, uric acid **(D)**, albumin levels **(E)** in the BALF. Data are presented as mean ± SEM (8-10 mice per treatment group). *P < .05 compared with PBS-treated mice.

Mice were treated intranasally with a panel of allergen extracts and the response to challenge determined after 1 hour. Papain and trypsin also were tested as examples of cysteine and serine proteases, respectively. Strikingly, *Alternaria* treatment resulted in a robust early IL-33 release, which was not observed in heat-treated *Alternaria* or with any other allergens and/or proteases (Fig 1, *A*). The failure to detect IL-33 in response to these other allergens was not a consequence of them eliciting IL-33 release and subsequently cleaving the cytokine into immunologically undetectable fragments (data not shown). From Western blots of the BALF, we determined that released IL-33 was a full-length protein (Fig 1, *B*). There was no significant increase in inflammatory cells to the lung or BALF (see Fig E1, *A* in this article's Online Repository at www.jacionline.org), which suggests that IL-33 release was from lung resident cells. The source of IL-33 in the BALF of *Alternaria*-exposed mice appeared to be Type II epithelial cells because they stained strongly for IL-33 in control mice, whereas the number of these IL-33^+^ cells was significantly reduced 1 hour after *Alternaria* exposure (Fig 1, *C*). The release of preformed IL-33 into the airways in response to *Alternaria* was confirmed by the concomitant reduction of IL-33 levels in lung tissue (Fig E1, *B*).

Cellular damage results in lactate dehydrogenase release, and levels were modestly elevated in BALF in response to *Alternaria*, but, greater still after papain exposure, an enzyme that failed to elicit IL-33 release (Fig E1, *C*). Similarly, although the alarmin uric acid also was released in response to *Alternaria,* papain again induced greater release (Fig E1, *D*). Thus, taken together, the *Alternaria*-induced increase in IL-33 release appears to be independent of cellular damage. BALF albumin levels were significantly elevated by *Alternaria* but also by papain (Fig E1, *E*), which suggests that loss of epithelial barrier function alone and influx of extrapulmonary mediators also is not sufficient to account for the *Alternaria*-induced IL-33. As with IL-33, IL-1β was only detectable in *Alternaria*-exposed animals (Fig 1, *D*). Similarly, MMP-9 secretion was observed in *Alternaria* and to a lesser extent papain and cat dander–treated mice (Fig 1, *E*). Furthermore, serum mast cell protease (MCPT-1) levels also were significantly increased in response to *Alternaria* (Fig 1, *F*).

**Fig 2:**
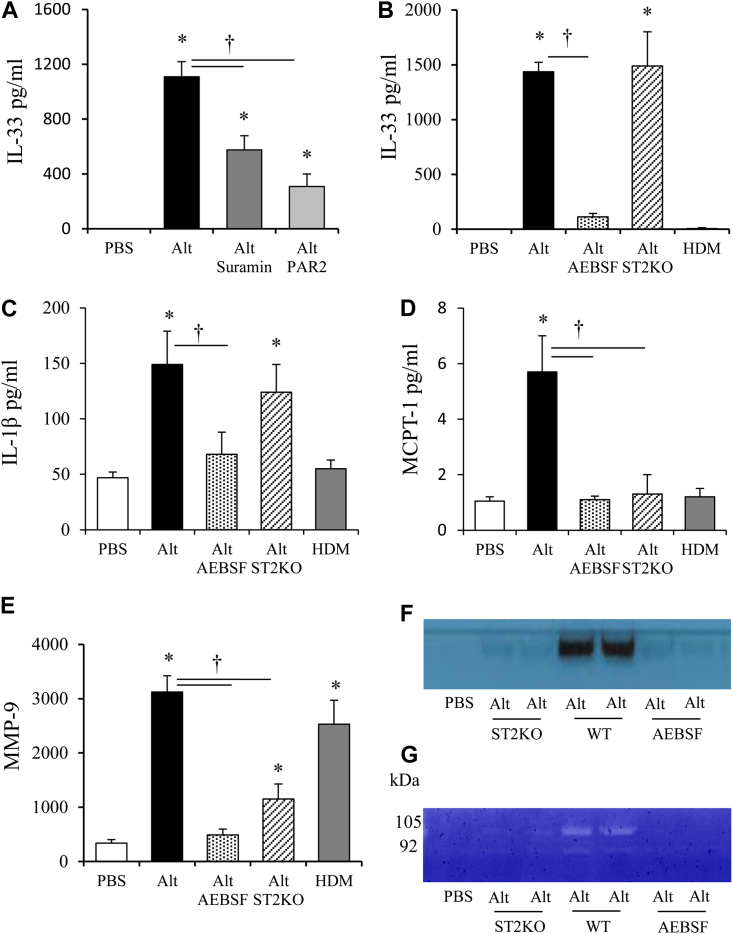
Serine proteases mediate innate mediator release. BALF IL-33 **(A** and **B)**, IL-1β **(C)**, and serum MCPT-1 **(D)** levels of mice treated with either HDM or *Alternaria (Alt)*. **E,** MMP-9 levels in the BALF. **F,** Western blot of MMP-9 and gelatin zymogram **(G)** for MMP-9 activity. Data are presented as mean ± SEM (8-10 mice per treatment group). Representative photomicrographs of gel images are shown. *P < .05 compared with PBS-treated mice. †*P* < .05 compared with *Alternaria*-treated mice.

*Alternaria*-induced IL-33 release *in vitro* has previously been reported to be ATP signalling dependent.^32^ Accordingly, blockade of ATP signaling *in vivo* through administration of P2 receptor antagonist suramin resulted in a 50% reduction in IL-33 release in response to *Alternaria* at 1 hour (Fig 2, *A*). Similarly, blocking PAR-2 receptor activation by endogenous proteases in the allergen inhibited IL-33 release into the BALF in response to *Alternaria* by 68% (Fig 2, *A*). The failure to completely abrogate IL-33 release by these inhibitors and/or antagonists may reflect redundancy within pathways. Because heating *Alternaria* abrogated the early innate mediator release, it may be mediated by an *Alternaria*-specific protease activity. The proteSEEKER (G-Biosciences, St Louis, Mo) assay was used to determine the relative protease activities present in different allergens. Cysteine protease activity was unique to HDM, whereas serine proteases also were present in *Alternaria*, cat dander, and ragweed (see Table E1 in this article's Online Repository at www.jacionline.org).

**Fig E2:**
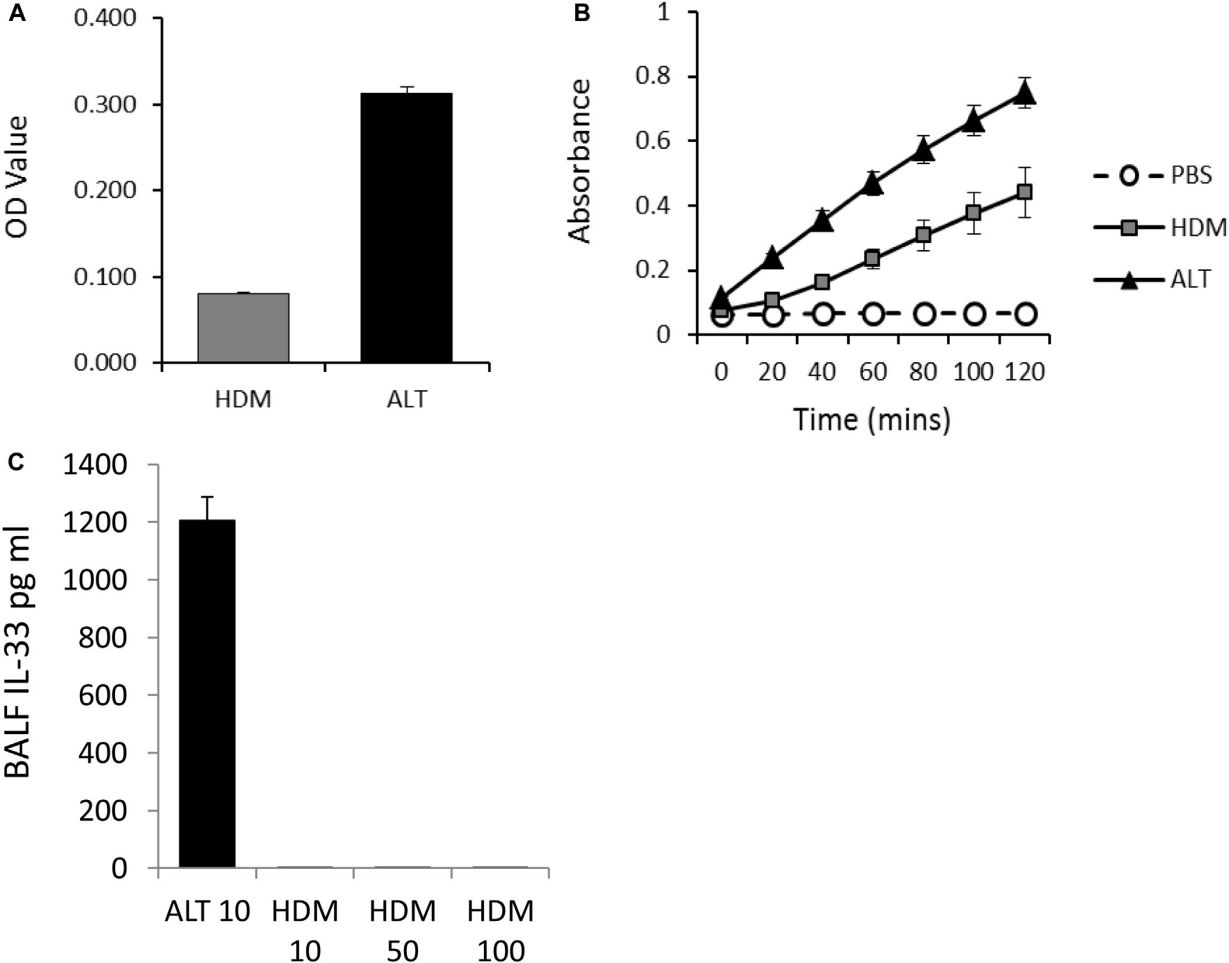
Protease activity of allergen extracts. **A,** Total protease activity of HDM and *Alternaria (ALT)*. **B,** Serine/cysteine protease activity determined by metabolism of the serine and cysteine protease substrate N-Benzoyl-Phe-Val-Arg-p-nitroanilide (NBP-VANA). Data are presented as mean ± SEM (n = 4). **C,** IL-33 levels in the BALF 1 hour after exposure to allergen. Data are presented as mean ± SEM (4-6 mice per treatment group).

The role of endogenous serine proteases in driving IL-33 release was determined by preincubating *Alternaria* with the serine protease inhibitor AEBSF before administration to mice. Intriguingly, the *Alternaria*-induced increase in IL-33 at 1 hour was almost completely dependent on serine protease activity (Fig 2, *B*). Because *Alternaria* exhibits slightly greater total and serine protease activity relative to HDM (see Fig E2, *A* and *B* in this article's Online Repository at www.jacionline.org), it is conceivable that the *Alternaria* specific IL-33 release is a function of greater protease activity of this allergen. To verify that this was not the case, it was demonstrated that even 10 times the dose of HDM failed to instigate IL-33 release (Fig E2, *C*). Furthermore, the *Alternaria*-induced increase in IL-1β, MCPT-1, and MMP-9 were ablated by AEBSF (Fig 2, *C*-*E*). The increase in MMP-9 release was confirmed by Western blot (Fig 2, *F*) and zymography (Fig 2, *G*). We subsequently determined whether IL-33 signaling through ST2 was critical for this innate mediator secretion by assessing their levels in *Alternaria*-exposed ST2KO mice. MCPT-1 (Fig 2, *D*) and MMP-9 release (Fig 2, *E*-*G*) were critically dependent on ST2 signaling and thus secondary to IL-33 release. In contrast, *Alternaria* induced IL-1β production was independent of ST2 signaling (Fig 2, *C*).

### *Alternaria*-derived serine protease activity drives early pulmonary cellular infiltrate

**Fig 3:**
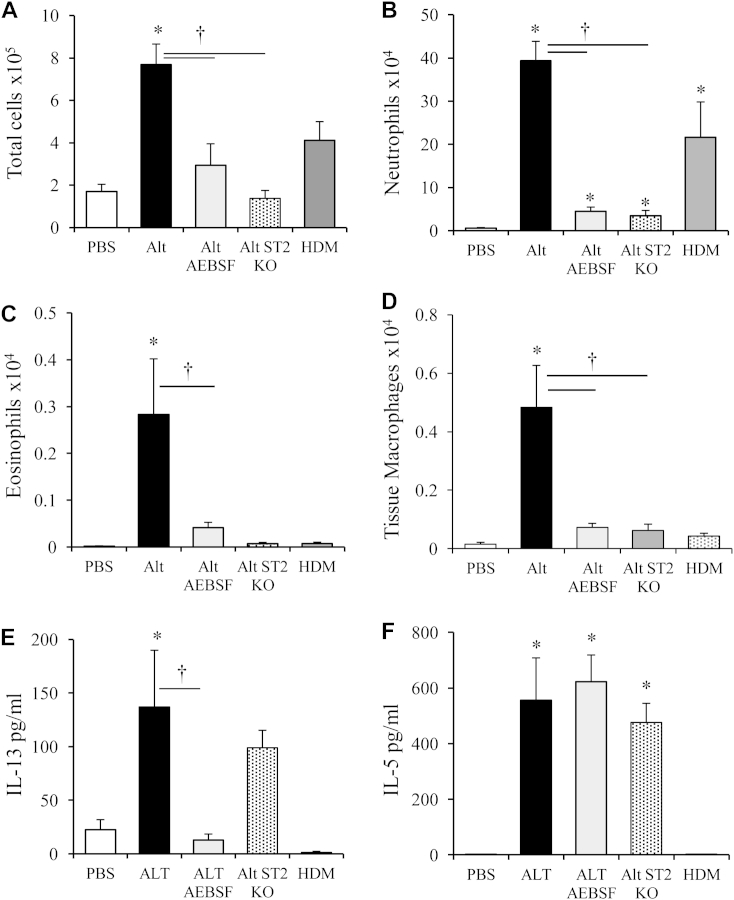
Cell recruitment to the lungs occurs within 24 hours. Mice were treated with either HDM or *Alternaria (Alt)*. Total cells **(A)**, neutrophils **(B)**, eosinophils **(C)**, tissue macrophages **(D)**, IL-13 **(E)**, and IL-5 **(F)** levels in the BALF. Data are presented as mean ± SEM (8-10 mice per treatment group). *P < .05 compared with PBS-treated mice. †*P* < .05 compared with *Alternaria*-treated mice.

To elucidate the role of *Alternaria* serine proteases and ensuing IL-33 release in driving early pulmonary inflammation, we assessed cellular recruitment at 24 hours in wild-type mice exposed to *Alternaria* with or without pretreatment with AEBSF, and *Alternaria*-exposed ST2KO mice. *Alternaria* exposure increased inflammatory cell infiltrate in the lung tissue (not shown) and BAL (Fig 3, *A*) at 24 hours. This infiltrate was predominantly neutrophilic (Fig 3, *B*), with an elevation of eosinophils and macrophages also apparent (Fig 3, *C* and *D*). Inhibition of endogenous allergen serine proteases or the absence of ST2 receptor significantly reduced neutrophilia, eosinophilia, and recruitment of tissue macrophages (Fig 3, *A*-*D*). In contrast, neutrophils were the only cell type elevated after HDM administration (Fig 3, *B*-*D*). IL-13 and IL-5 were elevated 24 hours after *Alternaria* administration (Fig 3, *E* and *F*), and independent of IL-33 signaling via the ST2 receptor. Interestingly, IL-13, but not IL-5, release was entirely dependent on serine proteases within *Alternaria*.

### *Alternaria*-derived serine protease activity exacerbates allergic airway disease

**Fig 4:**
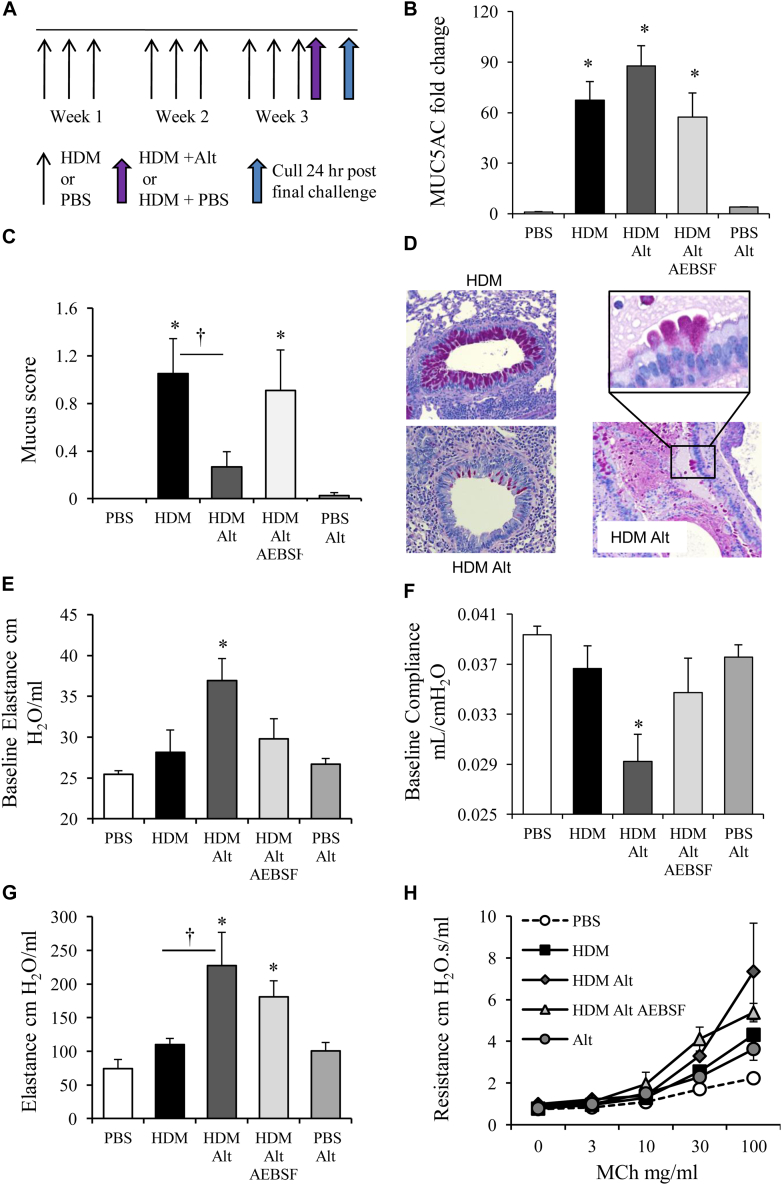
Exacerbation of HDM-induced allergic airway disease by *Alternaria*. Mice were treated with HDM and received a final challenge with *Alternaria (Alt)*. **A,** Schematic of exacerbation protocol. **B,** qPCR of MUC5AC transcript. **C,** Mucous score and periodic acid–Schiff staining of paraffin section, showing mucous (pink staining) **(D)**. **E,** Baseline elastance and baseline compliance **(F)**. **G,** Elastance at 100 mg/mL methacholine *(MCh)*. **H,** Airway resistance to increasing doses of methacholine. Data are presented as mean ± SEM (4-6 mice per treatment group). Representative photomicrographs are shown. Original magnification ×40. *Scale bar* = 50 μm. *P < .05 compared with PBS-treated mice. †*P* < .05 compared with HDM-treated mice.

**Fig E3:**
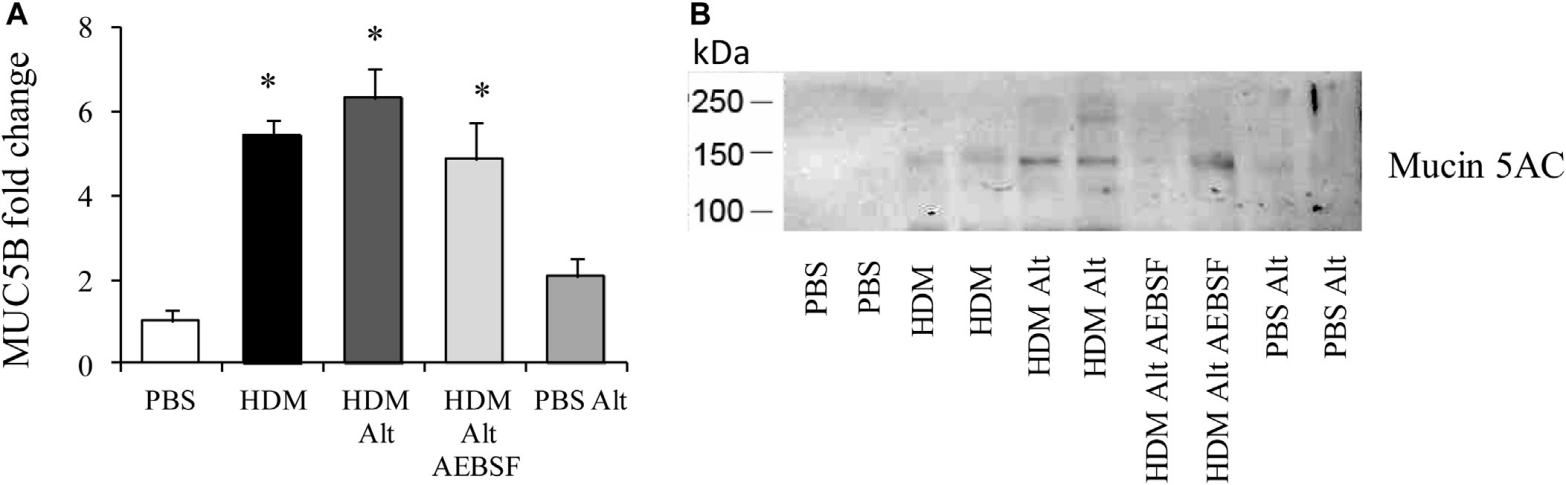
*Alternaria* induces mucus release in the exacerbation model. **A,** Fold increase in transcript for MUC5B determined by qPCR. **B,** Western blot for mucin 5AC in the BALF of mice exposed to HDM and subsequently treated with either *Alternaria* in the presence or absence of AEBSF or PBS. Data are 4-6 mice per treatment group. *P < .05 compared with PBS-treated mice.

Because *Alternaria* is associated with fatal asthma exacerbations, we developed a murine model of allergen-mediated exacerbation (Fig 4, *A*) to determine whether the capacity of *Alternaria* serine proteases to drive a robust inflammatory response contributed to a sudden enhancement in disease severity. Mice were treated with HDM for 3 weeks and then 24 hours before being culled were exposed to a single dose of *Alternaria*. HDM induces bronchiolar epithelial cells to change to a mucus-secreting phenotype (Fig 4, *B*-*D*).^33^ Accordingly, all HDM-treated groups showed an increase in MUC5AC and MUC5B message in lung tissue relative to controls (Fig 4, *B* and E3, *A* [in this article's Online Repository at www.jacionline.org]). However, mice previously treated with HDM for 3 weeks responded to *Alternaria* by releasing mucus into the airway lumen, which was dependent on the serine protease activity of the allergen (Fig 4, *C* and *D*). This was confirmed via a Western blot, which depicted increased MUC5AC in the BALF of HDM-*Alternaria* mice (Fig E3, *B*). The *Alternaria*-induced mucus secretion was associated with an increase in baseline elastance and a decrease in baseline dynamic compliance, which was abrogated by AEBSF treatment (Fig 4, *E* and *F*), which indicated that mucus plugging of the airways in response to this allergen increases the rigidity of the lungs and decreases their ability to expand and recoil. Similarly, methacholine-induced elastance was increased in the *Alternaria*-treated allergic mice (Fig 4, *G*). Airway resistance (a function of smooth-muscle contraction of the conducting airways) although increased was not significantly exacerbated by instillation of *Alternaria* (Fig 4, *H*).

**Fig 5:**
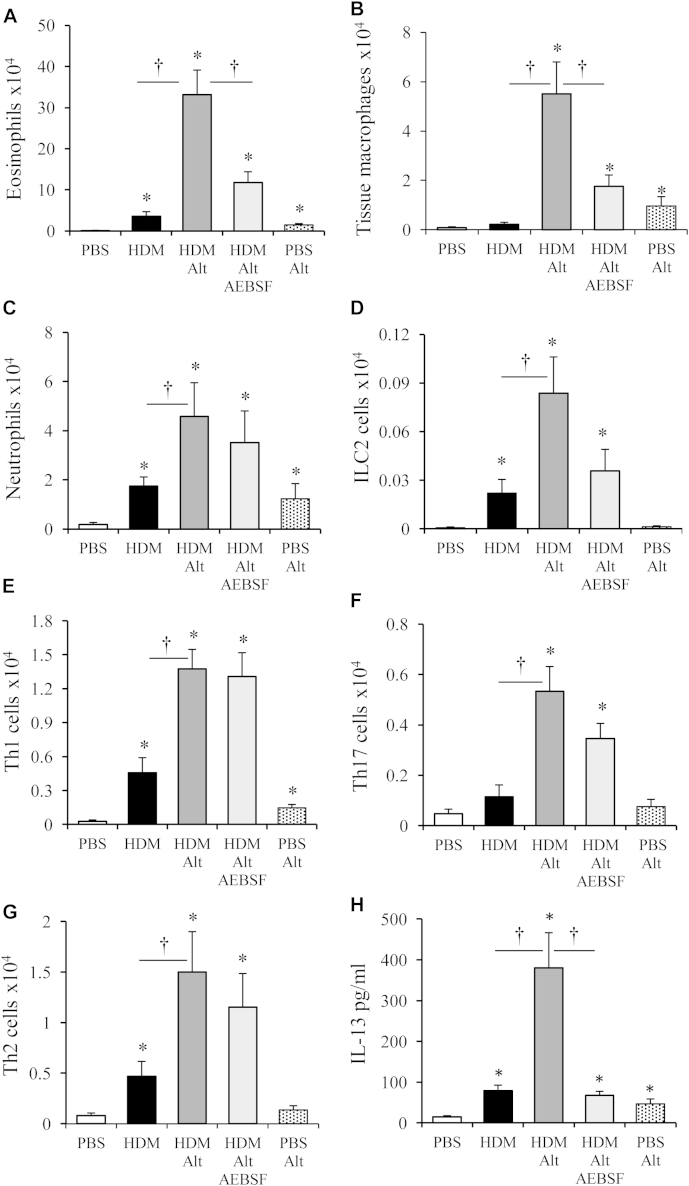
*Alternaria* results in inflammatory cell influx in allergic mice. Mice were treated with HDM and received a final challenge with *Alternaria (Alt)*. Eosinophils **(A)**, tissue macrophages **(B)**, neutrophils **(C)**, Lin^−^ IL-13^+^ ILC2 cells **(D)**, CD4^+^ IFN-γ^+^ T_H_1 **(E)**, CD4^+^ IL-17^+^ T_H_17 **(F)**, and CD4^+^ IL-13^+^ T_H_2 cells in the BALF **(G)**. **H,** IL-13 levels in the BALF determined by ELISA. *P < .05 compared with PBS-treated mice. †*P* < .05 compared with HDM or HDM ALT-treated mice. Data are presented as mean ± SEM (4-6 mice per treatment group).

Exposure of mice with HDM allergy to *Alternaria* resulted in a dramatic eosinophil influx into the airways (Fig 5, *A*). This eosinophilia was reduced through treatment with AEBSF, which implicates allergen-derived serine proteases in the exacerbated inflammation. Similarly, tissue macrophages (Fig 5, *B*) and neutrophils (Fig 5, *C*) were significantly elevated in the airways of *Alternaria*-exposed HDM-allergic mice. The increase in macrophages was partially dependent on *Alternaria* serine proteases (Fig 5, *B*), whereas the number of neutrophils was independent (Fig 5, *C*). ILC2 cells, which were increased after 3 weeks of HDM treatment, trebled in response to *Alternaria* challenge, an effect dependent upon serine protease activity (Fig 5, *D*). The influx of T_H_ cells was not reliant on proteases (Fig 5, *E*-*G*). However, IL-13 release, which was augmented in allergic mice, was completely dependent on protease activity (Fig 5, *H*).

## Discussion

Fungal allergens are a common cause of allergic asthma, with *Alternaria alternata* implicated not only in the development and persistence of asthma but specifically in rapid onset life-threatening exacerbations. We show that an *Alternaria*-specific serine protease activity elicits the rapid release of IL-33, which subsequently directs a robust early allergic inflammation and furthermore can significantly exacerbate disease in mice previously sensitized to a disparate allergen. This *Alternaria*-specific capacity to initiate the rapid and robust IL-33–dependent pulmonary allergic inflammation may define why patients with severe asthma exacerbations have frequently been exposed to *Alternaria*.

*Alternaria*-induced elevations in extracellular ATP and sustained augmentation of intracellular Ca^2+^ concentrations result in IL-33 release from airway epithelial cells.^32^, ^33^ Accordingly, we observed *Alternaria*-induced release of full-length IL-33 into the BALF of mice within 1 hour, which was dependent on ATP signalling. Importantly, we further defined the mechanism whereby *Alternaria* elicits IL-33 release, which demonstrates it to be dependent upon serine protease activity of the allergen and induced via activation of PAR-2. We propose a model whereby an *Alternaria*-specific protease activates PAR-2, which causes elevated extracellular ATP that, in turn, drives the release of preformed IL-33. This dependence of *Alternaria* intrinsic serine protease activity in driving this IL-33–mediated inflammation could underlie the potential of fungal proteases to act as T_H_2 adjuvants.^27^, ^28^ A recent study demonstrated that fungal-derived proteases cleave fibrinogen, which subsequently elicits allergic inflammation via TLR4 signalling,^29^ a mechanism that could also contribute to our observed *Alternaria*-specific phenotype. Furthermore, it is conceivable that crosstalk between TLR4-TRIF and PAR-2-TRIF signalling pathways drives allergic inflammation in response to *Alternaria,* as has been demonstrated in lipopolysaccharide models.^34^, ^35^

As with IL-33, early release of IL-1β was dependent on *Alternaria* serine protease activity and ATP signaling but did not lie downstream of IL-33 signaling. ATP is a known stimulus that drives the release of IL-1β,^36^, ^37^ and it would seem that an ATP-dependent pathway regulates release of IL-1 family proteins, IL-33 and IL-1β. Intriguingly, release of MCPT-1 and MMP-9 in response to *Alternaria* was not only dependent on intrinsic serine protease activity but was also secondary to IL-33 release. Both MCPT-1^38^ and MMP-9^39^, ^40^, ^41^, ^42^ are implicated in the development of allergic airway disease, although this is the first study to demonstrate that their release is IL-33 responsive.

We subsequently demonstrated that *Alternaria* elicited a rapid pulmonary inflammation by 24 hours after challenge, which was primarily neutrophilic, and was serine protease and ST2 dependent. A capacity of IL-33 to drive neutrophilic inflammation^43^, ^44^, ^45^, ^46^, ^47^ has been reported but not previously in the lung. *Alternaria* challenge also elicited an eosinophilic inflammation by 24 hours, not observed with HDM. The eosinophilia was dependent on serine protease activity of *Alternaria* and contingent on IL-33. The potential of IL-33 to promote IL-5^48^, ^49^ and CCL24^8^ release could contribute to this observed eosinophilia. This unique capacity of the *Alternaria* serine protease–IL-33 axis to drive an early pulmonary inflammation is supportive of clinical data that describe the potential of *Alternaria* spore exposure to compromise respiratory status of individuals within 24 hours and the more rapid onset of severe disease.^50^

*Alternaria* is associated with sudden, severe exacerbations of asthma.^24^, ^25^ and, therefore, we developed an exacerbation model of allergic airway disease. The level of eosinophilia is seemingly a biomarker for asthma exacerbations.^51^, ^52^, ^53^, ^54^ Exposure of HDM- sensitized mice to *Alternaria* resulted in a dramatic augmentation of eosinophilic inflammation that was seemingly fungal intrinsic serine protease activity dependent and, therefore, potentially contingent on IL-33. *Alternaria* exacerbation also resulted in a pronounced increase in numbers of infiltrating macrophages and ILC2 cells that were found to be reliant on serine protease activity. In ovalbumin-induced models of allergic airways disease, ST2 has been shown to be dispensable for T_H_2 inflammation.^55^ It is feasible that the role for ST2 is pronounced in our studies because of the protease pathway unique to *Alternaria* that elicits the release of IL-33. Furthermore, allergen sensitization is mediated by alum in ovalbumin models, which skews the immune response to a T_H_2 phenotype potentially bypassing a dependence on the innate pro-T_H_2 cytokine IL-33 signaling via its receptor ST2, which drives disease development after inhaled allergen.

Allergic mice responded to *Alternaria* instillation by releasing mucus into the airway lumen, which again was dependent on the serine protease activity of the allergen. This elevated mucus release may be driven by the observed changes in the IL-33 responsive cytokine, IL-13. The mucus plugging of the airways and significantly elevated cellular infiltrate in *Alternaria*-exacerbated mice likely accounted for the observed reduction in baseline elastance and increase in compliance. Thus, our model of asthma exacerbation elicited by *Alternaria* challenge recapitulates many of the cellular, structural, and physiological manifestations observed clinically, and intrinsic to these dramatic alterations is the aeroallergen serine protease activity and presumably its capacity to drive IL-33 release.

This study significantly augments our understanding of the pathogenicity of fungal aeroallergens. Specifically, we showed (1) *Alternaria*-driven release of IL-33 was attributable to a serine protease activity specific to this aeroallergen, (2) MMP-9 and MCPT-1 are novel downstream targets of IL-33, (3) the *Alternaria* intrinsic serine protease–IL-33 axis elicits a robust, rapid inflammation that may underlie the capacity of fungal proteases to act as T_H_2 adjuvants, and (4) the propensity of *Alternaria* serine protease activity to drive IL-33 release and ensuing inflammation rationalizes the clinical relevance of this aeroallergen at inducing severe rapid, onset asthma exacerbations. Given the increasing prevalence of fungal aeroallergens, it may be prudent to target this protease-IL-33 axis therapeutically in these instances of severe and exacerbated asthma.

**Key messages**

• An *Alternaria*-specific serine protease activity, which is lacking from other common aeroallergens, drives the robust early release of IL-33.
• This serine protease–IL-33 axis promotes pronounced pulmonary inflammation and innate mediator release.
• The rapid influx of innate lymphoid cells and eosinophils and mucus release into the airways in the allergic lung underlies the *Alternaria*-specific rapid exacerbation of allergic airway disease and decrease in lung function associated with severe asthma and fatal asthma attacks.

## Figures and Tables

**Table E1:** Allergen extracts (1 mg/mL HDM, 100 μg/mL all other allergens) were screened by using 8 protease inhibitors (ProteSEEKER assay) to determine protease classes present in the extracts∗

Inhibitor	HDM	ALT	CAT	RAG
Bestatin				
Aminopeptidases, exopeptidases	0	6.5	2.8	0
E-64				
Cysteine proteases	**24.4**	0	7.8	2.1
EDTA				
Metalloproteases	0	1.6	2.3	0
Leupeptin				
Serine and cysteine proteases	17.9	8.9	**22.9**	**24.2**
Pepstatin				
Aspartic proteases	0	5.7	**21.1**	0
AEBSF				
Serine proteases	11.5	**26.0**	**33.5**	17.9
Phosphoramidon				
Thermolysin, collagenase, metalloendoproteinases	6.4	**24.0**	11.0	12.6
Phenylmethanesulfonylfluoride (PMSF)				
Serine and cysteine proteases	0	**35.0**	**28.4**	**87.4**

∗ Results represent % inhibition of protease activity.

## Measurement of airway hyperresponsiveness

Measurements of dynamic resistance and compliance were performed by using the Flexivent system (Scireq, Montreal, Quebec, Canada). After induction of anesthesia with an intraperitoneal injection of pentobarbitone (50 mg/kg) (Sigma-Aldrich) and intramuscular injection of ketamine (200 mg/kg) (Fortdodge Animal Health Ltd, Southampton, United Kingdom), mice were tracheostomized and connected to the Flexivent ventilator via a blunt-ended 19-gauge needle. Mice were ventilated by using the following settings: tidal volume of 7 mL/kg body weight, 150 breaths/minute; positive end-expiratory pressure approximately 2 cm H_2_O. Standardization of the lung volume history was done by performing 2 deep inflations. Subsequently, measurements of airway function were made. Measurements of resistance, elastance, and compliance were determined from a user-defined protocol by using the snapshot-150 perturbation, which is a single-frequency sinusoidal waveform. Resultant data were fitted by using multiple linear regression to the single compartment model to determine the above parameters. The resultant data was fitted to the Constant-Phase model to determine the above parameters. Changes in lung-airway function parameters were measured in response to increasing concentrations of nebulized methacholine from 3-100 mg/mL, delivered into the inspiratory line of the flexivent ventilator. Airway resistance was expressed as cmH_2_O/mL/s, elastance was expressed as cmH_2_O/mL, and compliance was expressed as mL/cmH_2_O.

### Collection and preparation of samples

BALF was collected by lavage of the lungs 3 times with 0.4 mL of PBS via a tracheal cannula. BALF was centrifuged (700*g*, 5 minutes, 4°C), and cells were resuspended in 0.5 mL complete media (RPMI + 10% FCS, 2 mM L-glutamine, 100 U/mL penicillin/streptomycin). BALF cell supernatants were removed and analyzed for mediators by ELISA. To disaggregate the cells from the lung tissue, 1 lobe (approximately 100 mg) of lung was incubated at 37°C for 1 hour in digest reagent (0.15 mg/mL collagenase type D, 25 μg/mL DNase Type I) in complete RPMI media. The recovered cells were filtered through a 70-μm nylon sieve, washed twice, resuspended in complete media, and counted in a hemocytometer before FACS analysis.

### Assessment of airway remodeling

Goblet cells were visualized on periodic acid–Schiff stained lung sections and scored with results expressed as a mucus score in arbitrary units.E1, E2 Periodic acid–Schiff stained goblet cells in airway epithelium were measured double-blind by using a numerical scoring system (0, <5% goblet cells; 1, 5% to 25%; 2, 25% to 50%; 3, 50% to 75%; 4, >75%). The sum of airway scores from each lung was divided by the number of airways examined, 20 to 50 airways per mouse, and expressed as mucus cell score in arbitrary units.

### FACS

Single-cell suspensions were stained with the LIVE/DEAD Fixable Near-IR-Dead Cell staining kit (Molecular Probes, Invitrogen) for 10 minutes in PBS. Cells then were washed in PBS and stained for surface markers for 30 minutes at 4°C in PBS that contained 0.1% (wt/vol) sodium azide and 1% (wt/vol) BSA and were fixed with 2% (vol/vol) paraformaldehyde. Cell types were characterized according to their forward- and side-scatter profiles and by their phenotype as follows: neutrophils, Ly6G^hi^CD11b^hi^CD11c^−^F4/80^lo^; eosinophils, SiglecF^hi^Ly6G^lo^CD11b^hi^; alveolar macrophages, CD11b^neg-lo^CD11c^+^F4/80^hi^; tissue macrophages, CD11b^hi^CD11c^−^F4/80^hi^; T lymphocytes, CD3^+^CD4^+^ or CD3^+^CD8^+^; ILC2s, Lin^−^CD45^+^ICOS^+^.

For the detection of intracellular cytokines, 1 × 10^6^ cells per mL were incubated for 3 hours at 37°C with phorbol 12-myristate 13-acetate (50 ng/mL), ionomycin (500 ng/mL; Calbiochem, Nottingham, United Kingdom) and brefeldin A (10 mg/mL). Cells then were stained with anti-CD4 and anti-CD8 (for T lymphocytes) or anti-Lin, anti-CD45, and anti-ICOS (for ILC2s) and fixed as described above. After being made permeable for 10 minutes with a buffer of 1% (wt/vol) saponin, 1% (wt/vol) BSA, and 0.05% (wt/vol) azide in PBS (saponin buffer), cells were stained with anti-IFN-γ, anti-IL-17, and anti-IL-13 diluted 1:50 in saponin buffer. Then, 30 minutes later, cells were washed once in saponin buffer and once in PBS that contained 0.1% (wt/vol) sodium azide and 1% (wt/vol) BSA, and data were acquired on a Fortessa, which collected 5 × 10^4^ lymphocyte events that were analyzed with FACSDiva software (BD Bioscience).

### MMP-9 zymogram

BALF was mixed with an equal volume of Novex Tris Glycine SDS sample buffer (Invitrogen) and electrophoresed on Novex 10% zymogram (gelatine) gels (Invitrogen) at 125 V for 90 minutes. After electrophoresis, gels were incubated in Zymogram Renaturing Buffer (Invitrogen) for 30 minutes at room temperature. To visualize the gelatinolytic activity, the gel was incubated in Zymogram Developing Buffer (Invitrogen) overnight at 37°C before staining with SimplyBlue Safestain (Invitrogen).

### RNA extraction and real-time PCR

Total RNA was extracted from 50 to 100 mg of lung tissue by using the Qiagen RNeasy Mini Kit (Qiagen, Manchester, United Kingdom); cDNA was synthesized from 1 μg of total RNA prepared with the Moloney Murine Leukemia Virus (M-MLV) reverse transcriptase mix (Promega, Madison, Wis) and analyzed by means of PCR on a CFX96 real-time PCR detection system (Bio-Rad Laboratories, Hemel Hempstead, United Kingdom). Reactions were run in triplicate with TaqMan probes (Applied Biosystems, Invitrogen) and TaqMan Fast Advanced MasterMix (Applied Biosystems, Invitrogen). The expression of the MUC5AC (Applied Biosystems probe Mm0127626_g1) and MUC5B gene (Applied Biosystems probe Mm00466391_m1) was normalized against that of hypoxantine phosphoribosyltransferase (probe Mm01545399_m1; Applied Biosystems).

### Western blot

All the samples were electrophoresed through reducing SDS-polyacrylamide gels and were electroblotted onto nitrocellulose membranes. Membranes were blocked in TRIS-buffered saline solution that contained 5% milk for 1 hour at 22°C and were incubated overnight at 4°C with primary antibody to Muc5AC (2-11M1;1 μg/mL; Abcam, Cambridge, United Kingdom) diluted in TRIS-buffered saline solution that contained 5% milk. Once washed, the membranes were incubated with goat anti-mouse IgG-horseradish peroxidase (1/5000; Invitrogen), and reactivity was detected by using enhanced chemiluminescence (ECL) chemiluminescent kits (Pierce, Rockford, Ill) according to manufacturer's directions.
